# Effect of Acute Dietary Nitrate Supplementation on the Changes in Calf Venous Volume during Postural Change and Skeletal Muscle Pump Activity in Healthy Young Adults

**DOI:** 10.3390/nu16111621

**Published:** 2024-05-26

**Authors:** Anna Oue, Yasuhiro Iimura, Yuichi Miyakoshi, Masako Ota

**Affiliations:** Faculty of Health and Sports Sciences, Toyo University, 1-7-11, Akabanedai, Kita-ku, Tokyo 115-8650, Japan; iimura581@toyo.jp (Y.I.); miyakoshi@toyo.jp (Y.M.); masako@toyo.jp (M.O.)

**Keywords:** beetroot juice, nitric oxide, venous blood pooling, venous return

## Abstract

Dietary nitrate (NO_3_^−^) supplementation is known to enhance nitric oxide (NO) activity and acts as a vasodilator. In this randomized crossover study, we investigated the effect of inorganic NO_3_^−^ supplementation on the changes in calf venous volume during postural change and subsequent skeletal muscle pump activity. Fifteen healthy young adults were assigned to receive beetroot juice (BRJ) or a NO_3_^−^-depleted control beverage (prune juice: CON). Two hours after beverage consumption, the changes in the right calf volume during postural change from supine to upright and a subsequent right tiptoe maneuver were measured using venous occlusion plethysmography. The increase in calf volume from the supine to upright position (total venous volume [VV]) and the decrease in calf volume during the right tiptoe maneuver (venous ejection volume [Ve]) were calculated. Plasma NO_3_^−^ concentration was higher in the BRJ group than in the CON group 2 h after beverage intake (*p* < 0.05). However, VV and Ve did not differ between CON and BRJ. These results suggest that acute intake of BRJ may enhance NO activity via the NO_3_^−^ → nitrite → NO pathway but does not change calf venous pooling due to a postural change or the calf venous return due to skeletal muscle pump activity in healthy young adults.

## 1. Introduction

Nitric oxide (NO) plays a major role as a vasodilator and contributes to the regulation of blood pressure (BP) and blood flow in each tissue [1]. Generally, the enzymatic NO synthase pathway, which catalyzes the oxidation of _L_-arginine to NO and L-citrulline, is known as the classical NO production pathway [2]. In addition, NO can also be created by the stepwise reduction of nitrate (NO_3_^−^) and nitrite (NO_2_^−^) (i.e., the NO_3_^−^ → NO_2_^−^ → NO pathway) [3]. Many studies have used dietary NO_3_^−^ supplementation with beetroot juice (BRJ) to investigate the effect of the NO_3_^−^ → NO_2_^−^ → NO pathway. The increased NO activity induced by this pathway has been suggested to attenuate sympathetic nerve activity [4] and might act as a vasodilator [5,6,7,8,9,10,11,12], thereby contributing to the regulation of BP and vascular tone in various physiological conditions.

A postural change from supine to upright causes a translocation of blood from the upper body and thorax to the lower body due to the hydrostatic effects of gravity, which results in a reduced venous return and cardiac preload. However, because these decreases will be counterbalanced by some compensatory responses, BP and cardiac output (CO) are actually maintained. For example, sympathetic activation is enhanced via the unloading of cardiopulmonary and arterial baroreceptors [13,14]. In addition, skeletal muscle pump activation can also boost the venous return, with the skeletal muscles of the leg serving as an effective pump, driving venous blood back to the heart in combination with competent venous valves [15]. Because the heart has a pumping function that ejects blood but lacks the capacity to retain it, it is very important for CO and BP to be maintained to adequately return blood from the veins to the heart. However, it is not clear how the venous vascular response during postural change and subsequent skeletal muscle pump activity is influenced by the enhanced NO activity induced via the NO_3_^−^ → NO_2_^−^ → NO pathway. In order to maintain this vital activity in humans, it is essential for each tissue to be properly supplied with nutrients and oxygen, and BP must be maintained within a certain range. Under conditions in which large fluid shifts occur, since BP varies rapidly, it is very important to understand the effect of NO activity on venous vascular response, which is involved in BP regulation.

Thus, the purpose of this study was to clarify the effect of acute dietary NO_3_^−^ supplementation with BRJ on venous blood pooling due to a postural change and the subsequent venous return due to skeletal muscle pump activity. The hypothesis of our study was that the venous blood pooling and venous return amounts would be higher in a BRJ intake group than in a control beverage group because dietary NO_3_^−^ supplementation with BRJ attenuates sympathoexcitation [4] and enhances vasodilation [5,6,7,8,9,10,11,12].

## 2. Methods

### 2.1. Participants

Fifteen healthy individuals (9 males, 6 females; 22.1 ± 1.5 years; 166.7 ± 9.5 cm; 65.8 ± 15.1 kg) volunteered for this study. All females were not using oral contraceptives and all participated in the experiments in the self-assessed follicular phase (3–10 days after the start of menstruation). This study was approved by the Human Ethics Committee of Toyo University (TU2019-018-TU2020-H-019; Approval Date: 9 November 2020) and was carried out in accordance with the Declaration of Helsinki. Participants were informed of the purpose, procedures, and risks of this study prior to providing written and verbal informed consent. The participants were prohibited from alcohol and caffeine consumption and severe exercise for 24 h before each experiment, as well as the consumption of high-NO_3_^−^ foods (e.g., green leafy vegetables and traditional Japanese foods) for 3 days prior to each experiment [16,17]. Moreover, since oral bacteria influence reducing NO_3_^−^ to NO_2_^−^ in vivo [18], the participants abstained from using mouthwash.

### 2.2. Intervention

A NO_3_^−^-rich beverage (BRJ 140 mL/day; ~8 mmol NO_3_^−^; Beet It, James White Drinks, Ipswich, UK) and NO_3_^−^-depleted control beverage (CON; prune juice 166 mL/day, <0.01 mmol NO_3_^−^; Sunsweet prune juice, POKKA SAPPORO Food & Beverage, Ltd., Nagoya, Japan) were used with reference to previous studies [19,20]. The amount of prune juice (166 mL/day) was matched to the energy in 140 mL/day of BRJ.

### 2.3. Experimental Design

The participants visited our laboratory for a total five days. Firstly, they were familiarized with the experimental procedure and device. The main experiments were conducted on the second and third days. In addition, blood samplings were carried out on the fourth and fifth days.

### 2.4. Measurements Procedures

Systolic blood pressure (SBP), diastolic blood pressure (DBP), and mean arterial pressure (MAP) were measured continuously and noninvasively from the left middle finger by the Finapres NOVA (Finapres Medical Systems BV, Enschede, The Netherlands). In addition, stroke volume (SV), heart rate (HR), and CO were determined from the BP waveform using the Modelflow software program (Finapres Medical Systems BV). The average values for the last 3 s during the supine and standing positions before the tiptoe exercise were used as the supine and upright data, respectively.

**Figure 1:**
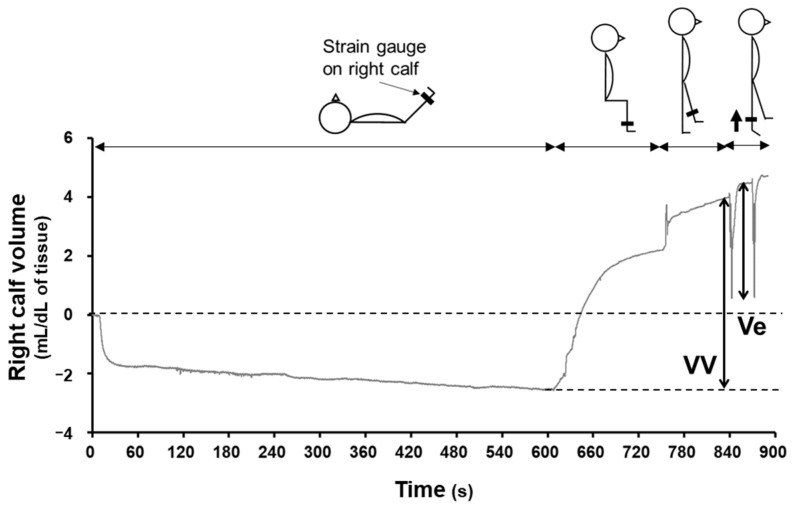
Diagrammatic representation of a typical plethysmography recording. Firstly, participants lay in a supine position with both legs elevated and sat down, stood up with the weight on the non-examined left leg, and carried out a single tiptoe maneuver. The changes in right calf volume during the leg lifting (both legs), sitting, standing, and tiptoe exercise sequence were measured. The increased venous volume due to the postural change (VV) and the ejection volume of the tiptoe exercise (Ve) were calculated.

To assess blood pooling and skeletal muscle pump activity, a strain gauge was attached at the maximal circumference of the right calf, and the change in the volume in the right calf was measured by using venous occlusion plethysmography (Hokanson, EC6; D. E. Hokanson, Bellevue, WA, USA) following the main experimental protocol (Figure 1). The increase in calf volume from the supine to upright position was defined as the total venous volume (VV), which is an index of the volume of blood pooling within veins. The decrease in calf volume during the tiptoe maneuver was defined as the ejection venous volume (Ve).

### 2.5. Protocol of the Main Experiment

Participants were instructed to consume CON or BRJ in a randomized crossover design. After their arrival at our laboratory, the participants ingested CON or BRJ. After 2 h, the participants were kept at rest in the supine position for 20 min before the recording of data in the laboratory (26.4 °C ± 0.5 °C). Skeletal muscle pump testing [21] was conducted after the participants rested in the supine position (Figure 1). Both legs were elevated by an angle of 15°–30° for 10 min to empty blood within veins from the calf and observe the presumptive minimum calf volume. The participant was asked to sit down for 2.5 min and then stand up with a slightly flexed right knee and weight on the left leg for 1.5 min. Then, participants performed a single tiptoe maneuver for 1 s. This movement generally induced an empty calf. The single tiptoe maneuver was repeated twice. A representative recording of the change in the right calf volume is shown in Figure 1.

### 2.6. Blood Sampling Protocol

To assess the plasma NO_3_^−^ concentration, venous blood was drawn from an antecubital vein before and 2 h after the intake of CON or BRJ on a different day from the main experiments.

### 2.7. Statistical Analysis

Data are presented as the mean ± standard deviation. To compare the changes in SBP, DBP, MAP, HR, SV, and CO from supine to upright between BRJ and CON, a two-way analysis of variance (ANOVA) with repeated measures (condition × postural change) was performed. If the main effect of the condition (BRJ and CON), that of postural change (supine and upright), and/or an interaction effect were detected, a paired *t*-test was applied as post hoc analysis. A two-way ANOVA with repeated measures (condition × time) was applied to test the comparison of plasma NO_3_^−^ between BRJ and CON. If the main effect of the condition (BRJ and CON), that of time (before and after intake), and/or an interaction effect were detected, a paired *t*-test was used as post hoc analysis. In addition, a paired *t*-test was used to test the comparison of the muscle pump activity (Ve and VV) between CON and BRJ. Statistical significance was set at less than 0.05. SPSS version 27 (IBM Corp., Armonk, NY, USA) was used for all statistical analyses.

## 3. Results

Table 1 shows the circulatory responses in the supine and upright positions before the tiptoe exercise in the CON and BRJ groups. The postural change under both BRJ and CON induced similar elevations of SBP, DBP, MAP, and HR and a decrease in SV. A postural change effect was observed for these parameters (all *p* < 0.02), and post hoc testing revealed significant differences in these parameters between the supine and upright positions (all *p* < 0.05). On the other hand, these parameters were similar for CON and BRJ in the supine and upright positions. CO was not different between both positions. In addition, CO was similar for CON and BRJ in both positions.

Ve (CON: 3.91 ± 0.80 mL/dL of tissue; BRJ: 4.04 ± 1.01 mL/dL of tissue) and VV (CON: 5.95 ± 1.18 mL/dL of tissue; BRJ: 6.06 ± 1.36 mL/dL of tissue) were not different between CON and BRJ.

The ingestion of BRJ caused an elevation in the plasma NO_3_^−^ concentration (before 16 ± 6 mM; after 572 ± 116 mM; *p* < 0.05). However, the plasma NO_3_^−^ concentration was similar before and after the intake of CON (before 15 ± 7 mM; after 15 ± 7 mM). The plasma NO_3_^−^ concentration in BRJ was significantly higher than that in CON after each beverage intake (*p* < 0.05).

## 4. Discussion

In this study, we investigated the effect of acute dietary nitrate supplementation with beetroot juice on the changes in calf venous volume during postural change and the subsequent tiptoe maneuver. The new finding in our study was that total venous volume due to a postural change from supine to upright and the ejection venous volume during a tiptoe maneuver did not differ beetroot juice and control beverage intake groups even though the plasma nitrate concentration after the intake of beetroot juice increased. This result suggests that although acute dietary nitrate supplementation may improve nitric oxide activity via the nitrate → nitrite → nitric oxide pathway, it cannot be concluded that it enhances calf venous pooling after postural change and subsequent calf venous return by skeletal muscle pump activity in healthy young humans. This is the first study to investigate the effect of inorganic NO_3_^−^ supplementation on the calf venous vascular response under the physiological condition that blood transfer occurs due to the hydrostatic effect of gravity.

VV did not differ between BRJ and CON. This result indicates that blood pooling within a vein of the leg during postural change from supine to upright cannot be influenced by acute intake of BRJ, which did not support our hypothesis based on the evidence that elevation of NO activity via the NO_3_^−^ → NO_2_^−^ → NO pathway attenuates sympathetic nerve activity [4] and acts as a vasodilator [5,6,7,8,9,10,11,12]. We considered two reasons why the VV was similar for BRJ and CON. First, the increase in NO activity with BRJ could not attenuate the elevation in sympathetic nerve activity due to the unloading of low-pressure (cardiopulmonary) baroreceptors. Second, the myogenic response compensated for the BRJ-induced venodilation [22]. In a previous study, sympathetic nerve activity was attenuated by BRJ intake when sympathoexcitation due to the stimulation of high-pressure baroreceptors occurred during exercise with the pressor response [4]. Adequate control of blood flow is vital for delivering oxygen and nutrients to tissues, which relies on the maintenance of BP. The shift of blood to the legs during the postural change from supine to upright decreases the venous return and preload. These decreases could be partly compensated for by the elevation of sympathetic nerve activity due to the unloading of cardiopulmonary baroreceptors [13,14]. If this sympathetic hyperactivity is weakened by the elevated exogenous NO activity, it would be expected to be unable to compensate for a reduced venous return and preload, making it difficult to maintain BP. In addition, if the vasodilating effect of increased exogenous NO activity is enhanced and the venous blood pooling in the lower extremities is increased, a marked decrease in venous return and subsequent decreases in BP and CO may occur. Thus, from the viewpoint of the maintenance of biological homeostasis, the results obtained in this study are physiologically reasonable.

Ve was also not different between BRJ and CON. This result indicates that the calf venous return due to skeletal muscle pump activity during the tiptoe maneuver was not influenced by BRJ. Venous return with skeletal muscle pump activity might be determined by the amount of venous blood pooling and the contractile strength of skeletal muscle [23]. As mentioned above, because the VV did not differ between BRJ and CON, the amount of venous blood pooling in the calf is speculated to be similar for BRJ and CON. In addition, previous studies reported that the intake of nitrate did not influence maximal isometric knee torque [24,25,26,27] or hand grip force [28,29,30]. Based on these results, we speculate that BRJ had no effect on the contractile strength of skeletal muscle during the tiptoe maneuver. Therefore, because the calf venous blood pooling and the muscle contractile strength of skeletal muscle were not influenced by acute intake of BRJ, it is likely that Ve was similar for CON and BRJ in the present study. In addition, as well as the effect of nitrate supplementation in our study, acute intake of L-citrulline supplementation is also likely to increase NO activity but not muscle oxygenation and performance [31]. These findings indicate that the effects of exogenous supplementation on physiological responses and performance remain largely unknown and require further investigation.

This study has several limitations. First, our results were obtained from a small number of participants and might have been subjected a degree of sampling bias. Thus, in the present study, the results cannot be generalized, and the conclusion is applied to a limited extent to healthy young adults. Second, although the assessment of NO_2_^−^ concentrations is important since the conversion of NO_3_^−^ to NO_2_^−^ is essential for biological effects to be exerted [32], we did not measure NO_2_^−^ concentrations. However, since it is reported that a significant elevation of the plasma NO_3_^−^ level causes an elevation in the NO_2_^−^ concentration in healthy young adults [33,34,35], we believe that NO_2_^−^ concentration in the plasma was also increased after consumption of BRJ in this study.

## 5. Conclusions

In this study, we investigated the effect of acute dietary NO_3_^−^ supplementation with BRJ on the changes in calf venous volume during postural change from supine to upright and a subsequent tiptoe exercise. Our findings suggest that although acute intake of beetroot juice causes an elevation in the plasma nitrate concentration, it cannot be concluded that it increases the calf venous blood pooling due to a postural change and the subsequent calf venous return due to skeletal muscle pump activity in young healthy adults.

## Figures and Tables

**Table 1:** Circulatory responses in supine and upright positions before tiptoe exercise in CON and BRJ.

	Supine		Upright		ANOVA *p*
	CON	BRJ	CON	BRJ	
SBP, mmHg	134 ± 15	130 ± 14	146 ± 20 *	141 ± 21 †	Condition: 0.182 Postural: 0.020 Interaction: 0.776
					
DBP, mmHg	79 ± 11	76 ± 8	103 ± 14 *	100 ± 14 †	Condition: 0.310 Postural: 0.001 Interaction: 0.817
					
MAP, mmHg	101 ± 12	98 ± 8	122 ± 16 *	118 ± 17 †	Condition: 0.236 Postural: 0.001 Interaction: 0.881
					
HR, bpm	64 ± 14	61 ± 13	88 ± 15 *	88 ± 10 †	Condition: 0.601 Postural: 0.001 Interaction: 0.304
					
SV, mL	86.4 ± 18.6	84.6 ± 18.2	58.3 ± 9.5 *	56.6 ± 8.4 †	Condition: 0.469 Postural: 0.001 Interaction: 0.961
					
CO, L/min	5.5 ± 1.6	5.2 ± 1.3	5.2 ± 1.0	5.1 ± 1.1	Condition: 0.220 Postural: 0.143 Interaction: 0.268

Values are mean ± standard deviation for n = 15 (9 males, 6 females). SBP: systolic blood pressure; DBP: diastolic blood pressure; MAP: mean arterial pressure; HR: heart rate; SV: stroke volume; CO: cardiac output. * *p* < 0.05, significant difference between supine and upright in CON. † *p* < 0.05, significant difference between supine and upright in BRJ.

## Data Availability

The data presented in this study are available on request from the corresponding author due to ethical reasons.
